# Pharmacotherapy variability and precision medicine in neurocritical care

**DOI:** 10.3389/fneur.2025.1630163

**Published:** 2025-07-18

**Authors:** Sherif Hanafy Mahmoud, Maged Kharouba, Asma Aboelezz, Adham Elshamy, Ellen Gunn

**Affiliations:** Neurotherapeutics and Clinical Pharmacokinetics (Neuro-CPK) Laboratory, Faculty of Pharmacy and Pharmaceutical Sciences, University of Alberta, Edmonton, AB, Canada

**Keywords:** precision medicine, neurocritical care, pharmacotherapy, pharmacokinetics, pharmacodynamics

## Abstract

Pharmacotherapy variability is defined as the variability in drug response among and within individuals that is attributed to the inter and intra-individual differences in the action and disposition of drugs. Neurological and medical complications in neurocritical care contribute significantly to the overall disease prognosis. Pharmacological management plays a key role in managing many of those complications such as cerebral vasospasm, delayed cerebral ischemia, hyponatremia, infections, and seizures. However, pathophysiologic changes secondary to neurological and critical illnesses make the medical management of these patients challenging, contributing to pharmacotherapy variability. Interindividual differences in disease pathophysiology, altered organ function, systemic inflammation, hemodynamic instability, and common interventions employed in intensive care settings could alter the pharmacokinetics and pharmacodynamics of medications. The use of potentially ineffective treatments and suboptimal dosing of medications to manage patients can lead to poor outcomes as the understanding of the effect of neurological injury on the action and disposition of drugs is limited. This narrative review highlights the factors contributing to pharmacotherapy variability in neurocritical care, equipping clinicians with critical insights to refine patient management strategies. In conclusion, pharmacotherapy variability within neurocritical care introduces additional layers of complexity that may significantly contribute to therapy failure, adverse drug reactions, and setbacks in drug development. Understanding these variations is essential for identifying subpopulations that may derive the greatest benefit from specific therapies, representing a critical step toward achieving precision medicine in neurocritical care, ensuring the administration of the appropriate medication to the right patient at the correct dosage regimen.

## 1 Introduction

Pharmacotherapy is essential in managing complications within neurocritical care, where timely and targeted interventions are crucial for patient survival and recovery. Neurocritical care includes a range of acute neurological conditions, including subarachnoid hemorrhage (SAH), intracerebral hemorrhage (ICH), traumatic brain injury (TBI), ischemic stroke (IS), and status epilepticus (SE). These conditions often present with secondary complications such as cerebral edema, seizures, vasospasm, delayed cerebral ischemia (DCI), electrolyte abnormalities, venous thromboembolism, infections, hemodynamic instability, and altered organ functions. The pharmacological management of these complications is vital for improving patient outcomes. However, pathophysiologic changes secondary to neurological and critical illnesses make the medical management of these patients challenging, contributing to *pharmacotherapy* var*iability*. Interindividual differences in disease pathophysiology, altered organ function, systemic inflammation, hemodynamic instability, and common interventions employed in intensive care settings could alter the pharmacokinetics (PK) and pharmacodynamics (PD) of medications, potentially resulting in lack of efficacy or increased toxicity. The use of potentially ineffective or toxic treatments and suboptimal dosing of medications to manage patients may increase poor outcomes as the understanding of the influence of neurological injury on the action and disposition of drugs is limited. Therefore, it is essential to investigate factors affecting pharmacotherapy variability.

The aim of this review is to highlight the factors contributing to pharmacotherapy variability in neurocritical care, equipping clinicians with critical insights to refine patient management strategies. We summarized our research and other studies investigating the variability in pharmacotherapy and its link to patient outcomes. Understanding these variations is essential for identifying subpopulations that may derive the greatest benefit from specific therapies, representing a critical step toward achieving precision medicine in neurocritical care.

## 2 Pharmacotherapy variability

### 2.1 Definition and significance

Pharmacotherapy variability is defined as *the variability in drug response among and within individuals that is attributed to the inter and intra-individual differences in the action and disposition of drugs*. Variability arises from differences in drug PK (absorption, distribution, metabolism, and excretion) and pharmacodynamics (drug action) among patients. In neurocritical care, where patients often present with altered physiology due to severe neurological injury, systemic inflammation, altered organ function, or concurrent therapies, these differences become even more pronounced. Tables 1, 2 summarize the PK and PD alterations in neurocritical care, respectively.

**Table 1:** Pharmacokinetic alterations in neurocritical care.

Pharmacokinetic alterations	Description	Examples
Absorption		
Reduced gut motility (Gastroparesis)	Neurological injury, sedation, and opioid use can delay the GIT transit time, resulting in reduced and/or delayed drug absorption.	Erratic and delayed oral absorption of phenytoin, leading to subtherapeutic levels and poor seizures control (125, 205). Oral antimicrobials such as fluoroquinolones (e.g., ciprofloxacin or moxifloxacin) or antifungals (e.g., fluconazole) may have reduced absorption in patients with poor gut perfusion, potentially leading to inadequate eradication of infections (206, 207). Oral anticoagulants (e.g., apixaban, rivaroxaban) can have variable absorption. Delayed gastric emptying or altered gut pH can affect their bioavailability and impact the consistency of their anticoagulant effect (208). Phenytoin exhibits significant adsorptive binding to enteral feeding tubes, resulting in reduced bioavailability and systemic drug exposure (209).
Impaired perfusion	Hypotension and vasopressor use (e.g., catecholamines) can alter blood flow to the GIT, affecting drug absorption.	
Enteral feeding	Continuous enteral feeding can interact with drug formulations, altering their absorption.	
Vomiting and diarrhea	Vomiting and diarrhea are common in the ICU, potentially reducing drug bioavailability.	
pH changes	Frequent administration of PPIs or other acid-suppressing agents elevates gastric pH, which can consequently influence the ionization state and subsequent gastrointestinal absorption.	
Distribution		
Hypoalbuminemia	Critical illness often leads to decreased serum albumin levels, resulting in a higher free drug fraction, potentially increasing the pharmacological effect and the risk of toxicity, but also clearance.	Hypoalbuminemia increases the free fraction of phenytoin and valproic acid, necessitating the frequent monitoring for their free levels to prevent toxicity (210–212). Moreover, it increases risk of bleeding associated with warfarin administration (213). The distribution of hydrophilic antibiotics like beta-lactams (e.g., cefepime) can be significantly altered by increased extracellular fluid volume, leading to lower than expected concentrations (214).
Altered BBB permeability	Neurological injury can disrupt the BBB, affecting the penetration of drugs into the CNS.	
Increased extracellular fluid volume	Cerebral edema and systemic fluid overload can increase the V_d_ of hydrophilic drugs, potentially leading to lower plasma concentrations.	
Metabolism		
Hepatic dysfunction	Critical illness can lead to hepatic hypoperfusion and dysfunction, impairing the activity of CYP450 enzymes responsible for metabolizing many drugs.	Commonly administered neuro-ICU medications such as ASMs (e.g., phenytoin, carbamazepine, and valproic acid) and antimicrobials (e.g., macrolides) are known substrates to CYP enzymes. Therefore, hepatic dysfunction or drug interactions can significantly alter their metabolism and subsequently their plasma levels. Enzyme-inducing ASMs, like carbamazepine, can enhance the metabolism of CYP3A4 substrates, reducing their effectiveness (215, 216). Systemic inflammation exert an inhibitory effect on the expression and activity of several CYP450 isoforms (e.g., CYP3A4), leading to impaired metabolic clearance of their respective substrate medications, such as benzodiazepine sedatives (e.g., midazolam), opioid analgesics (e.g., fentanyl), calcium channel blockers (e.g., nimodipine), and certain ASMs (e.g., carbamazepine), potentially resulting in increased systemic exposure (217).
Systemic inflammation	Systemic inflammation can inhibit CYP enzyme activity.	
Drug interactions	Polypharmacy in neurocritical care increases the risk of drug–drug interactions.	
Genetic polymorphism	Individual genetic variations in CYP enzymes can lead to significant variability in drug metabolism rates.	
Excretion		
Acute kidney injury	AKI is a common complication in neurocritical care, reducing glomerular filtration rate and impairing the elimination of renally cleared drugs and their active metabolites.	Renally eliminated medications such as certain ASMs (e.g., levetiracetam, gabapentin, and pregabalin), antimicrobials (beta-lactams and vancomycin), anticoagulants (e.g., enoxaparin), are highly affected by fluctuations in kidney functions or renal perfusion such as in AKI or ARC, where clearance is significantly affected, requiring dose adjustments (24, 67, 218). Studies showed cefepime, vancomycin and levetiracetam are cleared by RRT, requiring dose optimizations for patients undergoing RRT (219–221). Therapeutic hypothermia has been found to reduce the clearance of various CYP450 substrates such as phenytoin, propofol, midazolam, pentobarbital. Another example is the accumulation of morphine due to the inhibition of UGT (222, 223). Moreover, hypothermia can inhibit transporter activity, affecting their substrates and altering their renal excretion.
Augmented renal clearance	ARC has been frequently seen in neurocritical care population, resulting in subtherapeutic levels of renally eliminated drugs.	
Changes in renal blood flow	Hypotension and vasopressor use (e.g., catecholamines) can alter renal perfusion, affecting drug excretion.	
Renal replacement therapy	Patients with severe AKI may require RRT, which can significantly remove certain drugs from the circulation.	
Therapeutic hypothermia	Therapeutic hypothermia induces a systemic reduction in both metabolic and excretory processes, consequently diminishing the functional capacity of CYP450 enzymes and drug transporters.	

AKI, acute kidney injury; ARC, augmented renal clearance; ASMs, antiseizure medications; BBB, blood brain barrier; CNS, central nervous system; CYP450: cytochrome p450; GIT, gastrointestinal tract; ICU, intensive care unit; PD, pharmacodynamics; PK, pharmacokinetics; PPI, proton pump inhibitors; RRT, renal replacement therapy; UGT, Uridine diphosphate-glucuronosyltransferase; V_d_, volume of distribution.

**Table 2:** Pharmacodynamic alterations in neurocritical care.

Pharmacodynamic alterations	Description	Examples
Altered drug sensitivity/toxicity			Patients with brain injuries or other neurological illnesses often exhibit altered pharmacodynamics. The involvement of the CNS can lead to significant pharmacotherapy variability due to several factors: *Receptor modulation*: neurological damage alters the expression of neurotransmitter receptors and drug targets within the CNS and peripheral nervous system. This can lead to diminished or enhanced drug efficacy. *Autonomic nervous system dysregulation*: this can alter physiological responses such as heart rate, blood pressure, and gastrointestinal motility. *Endothelial dysfunction and BBB disruption*: many neurological conditions are associated with endothelial dysfunction, which can compromise the integrity of the BBB. *Altered drug metabolism and elimination*: neurological conditions can influence drug elimination processes through systemic effects on organ function or altered blood flow.	Patients with brain injury or increased ICP may exhibit increased sensitivity of GABA_A_ receptors. Therefore, this might potentiate the sedative effects of benzodiazepines (e.g., lorazepam, midazolam), resulting in prolonged sedation and respiratory depression (224). Similar to sedatives, patients with neurological illnesses can be more sensitive to the respiratory depressant and sedative effects of opioids such as fentanyl and morphine (225). SAH patient exhibits increased sensitivity to exogenous vasopressin, leading to increased propensity to hyponatremia (3). In patients with status epilepticus or severe brain injury, the brain may become less responsive to doses of benzodiazepines. This can be due to changes in receptor expression (internalization of GABA_A_ receptors) (226). Certain medications, including some antidepressants (e.g., bupropion, tricyclic antidepressants) and antipsychotics (e.g., clozapine), exhibit proconvulsant effects by lowering the seizure threshold. This necessitates the need to reassess their use in such patient population and careful dose optimization of ASMs to maintain seizure control in susceptible individuals (227).

ASMs, antiseizure medications; BBB, blood brain barrier; CNS, central nervous system; GABA, Gamma-aminobutyric acid; SAH, subarachnoid hemorrhage.

This pharmacotherapy variability directly influences patient outcomes by affecting the balance between therapeutic efficacy and the risk of adverse events. For example, subtherapeutic levels of antiseizure medications (ASM) in SE could result in uncontrolled seizures, worsening neurological outcomes (1, 2). Another example is the increased risk for hyponatremia in SAH patients receiving exogenous vasopressin infusion due to increased sensitivity in this patient population (3).

Pharmacotherapy variability contributes to the heterogeneity of treatment effect observed in clinical trials within neurocritical care (4, 5). Variability in drug absorption, distribution, metabolism, and excretion driven by patient-specific factors such as organ dysfunction, genetic polymorphisms, disease severity, and concurrent therapies can lead to wide inter-individual differences in drug exposure and response. This variability can mask true drug effects or falsely attribute outcomes to the intervention, leading to inconclusive or misleading trial results. To illustrate, in studies investigating the effect of hypothermia combined with standard of care versus standard of care alone, hypothermia alone can reduce the clearance of many standard of care drugs, potentially leading to toxicity if doses are not adjusted accordingly. In other words, the standard of care ceases to be truly standard if variability in pharmacotherapy is not taken into account. Recognizing and accounting for pharmacotherapy variability is therefore essential for accurately interpreting trial findings, optimizing therapy, and advancing precision medicine in neurocritical care.

The economic implications of pharmacotherapy variability within neurocritical care settings have not been extensively studied. Nevertheless, the overall economic burden in these settings can be inferred by considering the implications of pharmacotherapy variability. Notable factors include adverse drug reactions, toxicity, and treatment failures, all of which contribute to treatment escalation and extended hospital stays. These outcomes significantly increase healthcare costs (6–8). Therefore, addressing pharmacotherapy variability and precision medicine could potentially reduce the economic impact and improve patient outcomes (9).

In summary, pharmacotherapy variability in neurocritical care is a critical factor that can significantly impact patient outcomes. Current clinical guidelines have not fully taken into consideration drug variability in neurocritical care. Therefore, understanding and addressing this variability is essential to optimize drug efficacy and minimize adverse effects.

### 2.2 Factors contributing to pharmacotherapy variability

**Figure 1:**
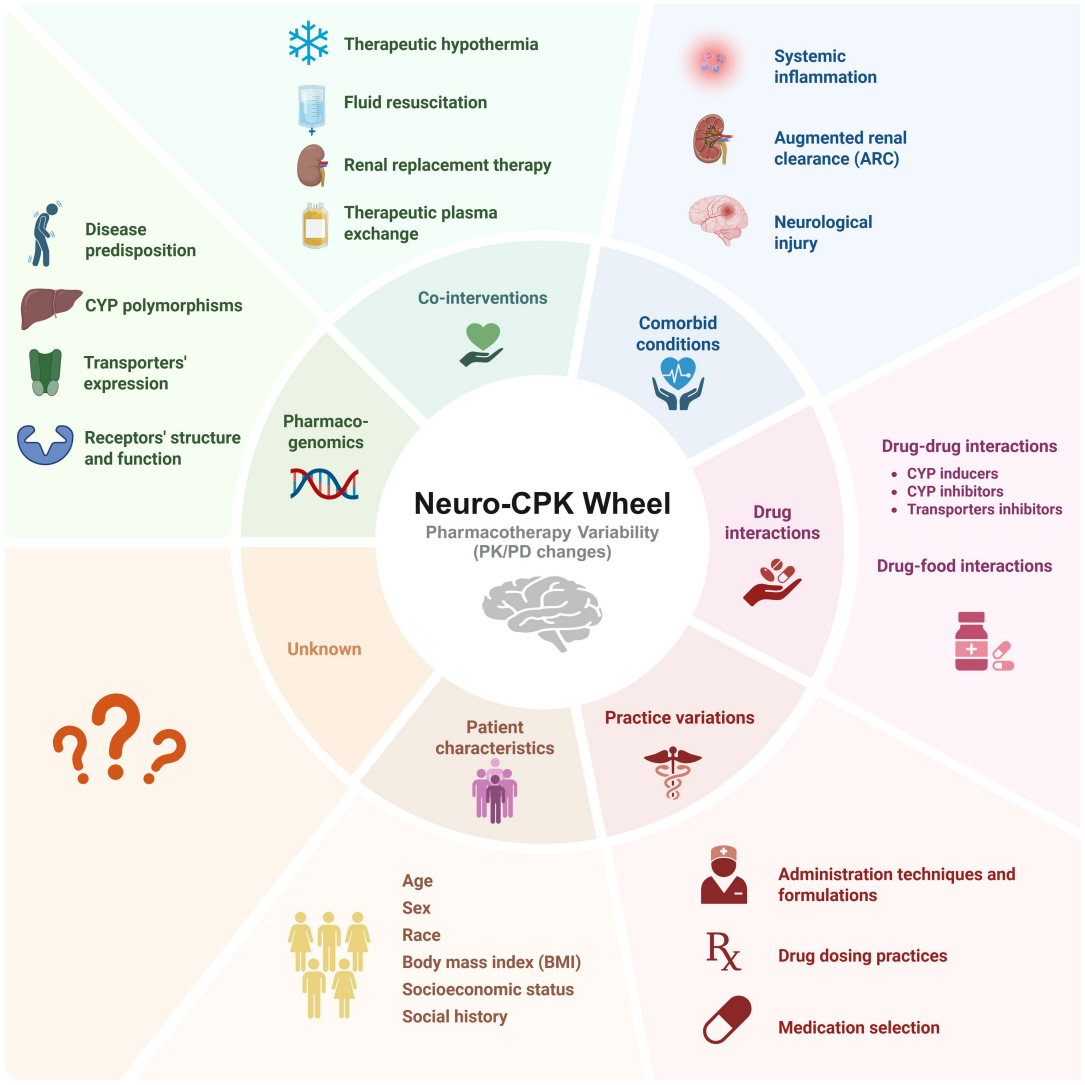
Hanafy’s *Neuro-CPK Pharmaco-variability Wheel* illustrates the factors influencing the pharmacokinetics (PK) and pharmacodynamics (PD) of drugs in neurocritical care. *Pharmacogenomics* contribute to inter-individual variability in drug responses, affecting PK through mechanisms such as cytochrome P450 (CYP) polymorphisms and altered transporter expression, and PD through drug tolerance and structural/functional changes at the drug-target level. *Co-interventions* in neurocritical care, including renal replacement therapies, therapeutic hypothermia, fluid resuscitation, and therapeutic plasma exchange, can result in pharmacotherapy variability. *Comorbid conditions* like systemic inflammation, ARC, and neurological injury impact the body’s drug response, leading to erratic and unpredictable drug levels. *Practice variations* between institutions and health care teams are critical factors often underemphasized. Different formulations and administration techniques result in variable drug plasma levels. Drug dosing practices may also vary. Concomitant medication administration is prevalent among ICU patients, resulting in *drug–drug interactions*, with many drugs classified as CYP inducers or inhibitors. Drug-food interactions may also occur through adsorption, chelation, or complexation. Patient characteristics, including age, sex, race and socioeconomic status are important factors affecting drug concentrations in the body. Despite efforts to identify factors causing drug variability, gaps remain that are currently unexplainable. Neuro-CPK, Neurotherapeutics and Clinical Pharmacokinetic laboratory; PK, pharmacokinetics; PD, pharmacodynamics; CYP, Cytochrome P450 enzymes. Created in BioRender. Lab, Neuro-CPK. (2025) https://biorender.com/bu0h3tj.

There are several factors that contribute to pharmacotherapy variability. Figure 1 illustrates *the Neuro-CPK Pharmaco-*var*iability Wheel.* It outlines the main factors affecting pharmacotherapy variability in neurocritical care, including comorbid conditions, drug interactions, practice variations, patient characteristics, pharmacogenomics, and co-interventions. Comorbid conditions could lead to physiological changes that affects how the body handle drugs (10). Moreover, drug interactions play a significant role in pharmacotherapy variability as neurocritical care patients usually receive multiple drugs (11, 12). Furthermore, variations in practice within the same health care facility or between different facilities could result in significant discrepancies in pharmacotherapy (13). Patient’s specific characteristics such as age, sex and body weight are major well-known factors contributing to variability (14). Additionally, monogenic and polygenic variability substantially contribute to the diverse PK and PD effects observed across individuals receiving the same medications (15). And finally, neurocritical care patients receive different co-interventions which create a highly variable environment for pharmacotherapy that may be overlooked in practice. In the following sections we discuss each factor in detail, providing examples and discussing the implications of these factors on pharmacotherapy variability. It is crucial to consider all potential factors that influence drug PK and PD, as the observed drug response in patients is essentially the result of the interplay among all these factor.

## 3 Drug-disease interaction (effect of comorbid conditions)

Drug-disease interactions are important factors affecting pharmacotherapy variability in neurocritical care. Critical illness and neurological conditions can significantly change drug PK and PD through systemic physiological changes, such as altered organ function (e.g., augmented renal clearance), systemic inflammation, hemodynamic instability, changed plasma protein levels, and disrupted blood–brain barrier integrity. These disease-induced changes can result in unpredictable subtherapeutic or toxic drug levels, and increased risk of adverse effects. Understanding drug-disease interactions is therefore essential for minimizing variability, optimizing individualized therapy, and improving outcomes in this highly vulnerable patient population.

### 3.1 Augmented renal clearance

Augmented renal clearance (ARC), a state of renal hyperfiltration, is a clinical phenomenon observed in critically ill patients (16). First described by Udy et al. (17) following observations of unexpectedly high creatinine clearance (CL_CR_) values during investigating antimicrobials PK in critical care population. ARC is primarily defined by elevated CL_CR_. However, the exact threshold remains debated. The most commonly cited definition utilizes CL_CR_ normalized to body surface area (BSA), with a threshold of >130 mL/min/1.73 m^2^ (16). ARC significantly enhances the elimination of renally excreted medications, potentially leading to subtherapeutic drug levels and compromised treatment outcomes. Notably, commonly used estimated CL_CR_ equations, such as Cockcroft-Gault, often underestimate ARC occurrence (18). This underestimation, coupled with the infrequent use of measured CL_CR_ in clinical practice, contributes to the frequent oversight of ARC. The reported prevalence of ARC varies considerably across studies, largely due to differences in the patient populations examined. A meta-analysis by Hefny et al. (19) indicated an overall ARC prevalence of approximately 36% in mixed intensive care unit (ICU) patients. However, the percentage rises significantly in neurocritical care, with reported prevalence of 74%, highlighting the significance of this phenomenon in neurocritical care.

**Figure 2:**
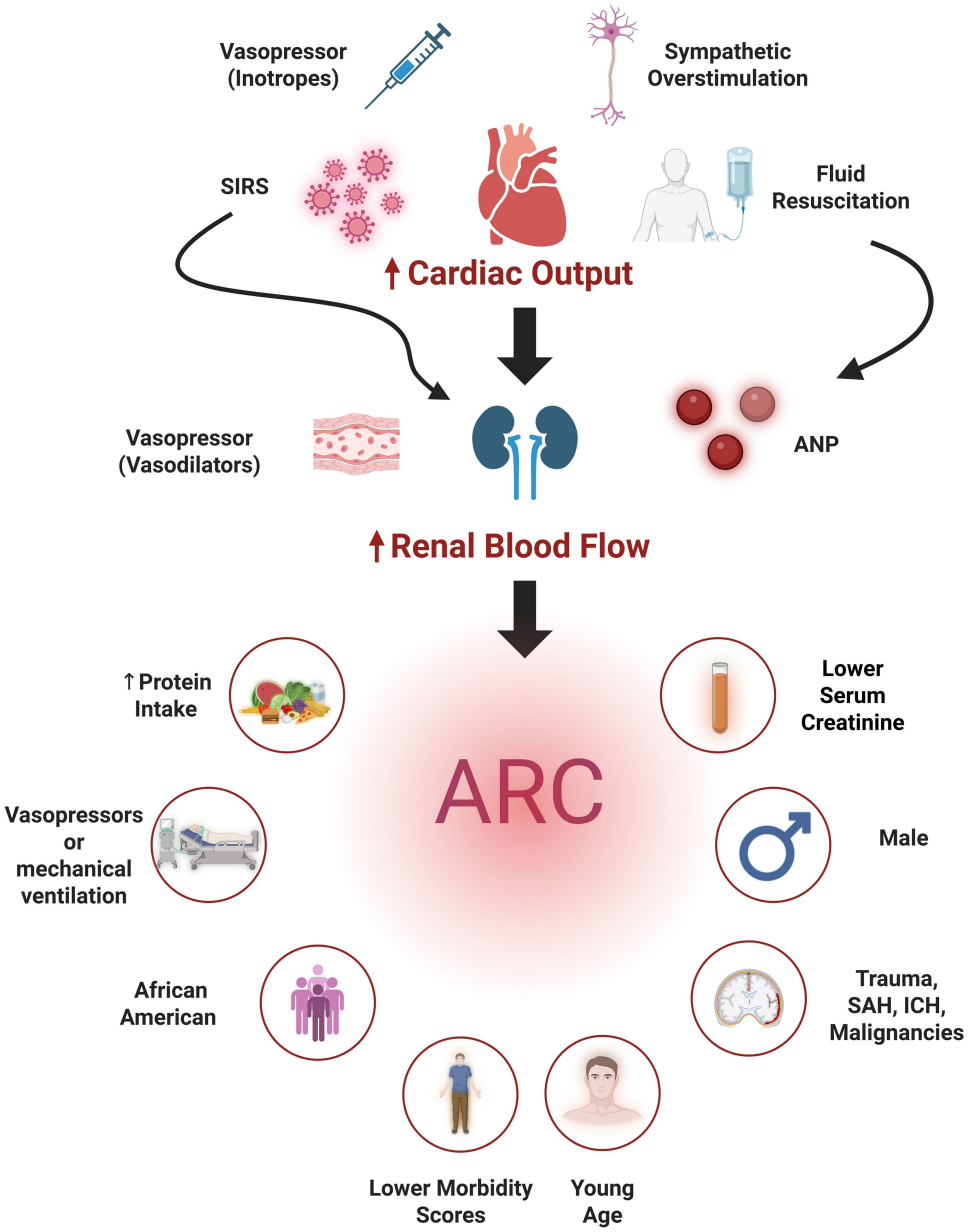
Pathophysiology of augmented renal clearance (ARC) (top) the use inotropes, autonomic dysregulation, fluid resuscitation, and systemic inflammatory response syndrome (SIRS) contribute to increasing cardiac output and hence renal blood flow. Other mechanisms (e.g., vasodilatory vasopressors, atrial natriuretic peptide (ANP) secreted secondary to hypervolemia, or SIRS directly increase renal blood flow), also contribute subsequently leading to ARC development. Independent predictors of ARC (bottom) comprise patient demographics (young age, male sex), neurological illnesses [traumatic brain injury, intracranial hemorrhage (ICH) and subarachnoid hemorrhage (SAH)], and clinical factors (e.g., lower morbidity scores, lower serum creatinine, increased protein intake, and vasopressor administration). Created in BioRender. Lab, Neuro-CPK. (2025) https://BioRender.com/0fuwuz5.

The pathophysiology of ARC remains incompletely understood (Figure 2). The hyperdynamic state characteristic of critical illness, driven by increased sympathetic response, increased renal blood flow, and the use of vasopressors or aggressive fluid resuscitation, contribute to ARC development. Emerging research also highlights the role of inflammatory mediators in augmenting kidney functions. Systemic inflammatory response syndrome (SIRS), common in critical care, has been implicated in increasing glomerular filtration rate (GFR) and subsequently inducing ARC (20). Specifically, atrial natriuretic peptide (ANP) has been identified as a potential mediator. Studies have demonstrated an association between elevated ANP levels and ARC development in patients with TBI (21). Regarding the underlying renal mechanisms, Udy et al. (22) demonstrated that ARC is characterized by concurrent increases in GFR, tubular reabsorption, and active tubular anion secretion. These combined effects result in enhanced excretion of renally cleared drugs.

Younger age, particularly below 50 years, and male sex are consistently identified demographic risks (17, 23–25), with a single study also identifying African American race as a risk factor (26). Other reported risk factors include trauma (24), hematological malignancies (27), ICH (28), the absence of cardiovascular comorbidities and lower morbidity scores (28–31). Low baseline serum creatinine concentrations (25), the use of mechanical ventilation and vasopressors (30, 32), and increased protein intake (26) were also reported as ARC risk factors. One study identified SAH, especially when accompanied by younger age, higher mean arterial pressure, absence of prior hypertension, and increased nitrogen loss significantly elevate ARC risk (33).

Current research focuses on optimizing drug dosing regimens in the context of ARC (34). Strategies to mitigate the risk of subtherapeutic drug exposure include escalating drug doses, shortening dosing intervals, administering drugs via continuous infusion, or prolonging infusion durations.

#### 3.1.1 Impact of ARC on renally eliminated antiseizure medications

Seizures are common following acute neurological illnesses, necessitating immediate initiation of adequately dosed ASMs (35–37). ARC can significantly increase the clearance of renally eliminated ASMs, potentially resulting in reduced drug exposure, therapeutic failure, and breakthrough seizures (24).

*Levetiracetam* is a first line ASM that is commonly used to control seizures in neurocritical care, with reference range of 12–46 mg/L. Levetiracetam is predominantly renally eliminated, with ~66% of the administered dose excreted unchanged in urine (24). The remaining portion is eliminated by non-cytochrome P450 enzymes, mainly by hydrolysis. Several studies have reported the positive correlation between CL_CR_ and levetiracetam clearance (38–40). Consequently, ARC significantly decrease levetiracetam exposure, which necessitates an increase in the dosage to achieve concentrations within the reference range (24, 41). The recommended initial dose of levetiracetam of 500 mg twice daily was clearly proven to result in subtherapeutic concentrations in patients with ARC, necessitating at least 1,500 mg to be administered twice daily to achieve concentrations within the reference range (24, 34, 42).

*Lacosamide* is utilized in the management of focal and generalized seizures, typically as adjunctive therapy or for seizures refractory to first-line agents and status epilepticus (43). Exhibiting linear PK, approximately 40% of lacosamide is eliminated unchanged via renal excretion (44). Studies have also demonstrated a positive correlation between CL_CR_ and lacosamide clearance in patients with renal impairment, highlighting the impact of renal function on drug elimination (44–46). There is a gap in the literature about ARC’s impact on lacosamide pharmacokinetics, more research is needed (47).

*Other antiseizure medications* such as pregabalin and gabapentin are commonly used in neurocritical care primarily for neuropathic pain; they are less frequently employed for seizure control in the ICU. Both drugs are predominantly eliminated by the kidneys, with almost 100% of gabapentin and approximately 90% of pregabalin excreted renally (48, 49). Their clearance strongly correlates with CL_CR_ (50–54). Further research is needed to establish appropriate administration strategies for these medications in this patient population. Topiramate is another medication less commonly used in the ICU. Research has demonstrated a correlation between its clearance and renal function (55). While studies have extensively investigated dose adjustments for reduced kidney function (48, 56, 57), the impact of ARC on ASM exposure in neurocritical care remains unstudied.

#### 3.1.2 Impact of ARC on renally eliminated antimicrobials

Neurocritical patients are highly prone to hospital acquired infections (HAI) due to prolonged ICU stay, invasive procedures and compromised neurological function (58). HAI occurs in approximately 11% of neurosurgical ICU patients, however, the rate increases to 36% for those who stays for more than 48 h (59). Examples of HAI include ventilator-associated pneumonia, catheter- associated urinary tract infection, central line- associated blood stream infections and surgical site infections (60). A wide range of antimicrobials are used to treat these infections, and the majority are renally eliminated (61). ARC may lead to sub-therapeutic-drug concentrations of renally eliminated antimicrobials, treatment failure and the development of antimicrobial resistance (17). It is essential to achieve therapeutic concentrations as early as possible because the time is a significant factor in infections, as for example each one hour delay in the start of antimicrobials may result in 9% increase in mortality in sepsis (62). Among the most used antimicrobials to treat HAI are vancomycin, piperacillin-tazobactam and meropenem. Around 60–90% of these antibiotics are excreted unchanged through the kidney, thus any alterations in the kidney function could have a crucial impact on their systemic exposure (63, 64).

*Vancomycin* is a glycopeptide antibiotic used to treat infections caused by gram positive bacteria especially those caused by methicillin resistant staphylococcus aurous (MRSA) (65). Therapeutic drug monitoring (TDM) is recommended for vancomycin. It is recommended to achieve a target area under the curve/minimum inhibitory concentration (AUC/MIC) ≥ 400 (66), or if difficult to obtain AUC, a trough concentration within the range of 10–20 mg/L is to be targeted as a surrogate measure. ARC patients on standard vancomycin dosing have been reported to achieve below-target trough concentrations, requiring higher doses (67–70).

*Piperacillin-tazobactam* is a broad spectrum *β*-lactam antibiotic that used to treat infections secondary to gram negative bacteria especially *Pseudomonas aeruginosa* (71). Since TDM of piperacillin is not a part of the standard care, ensuring target therapeutic concentrations is essential. It was found that around 37% of patients who have CL_CR_ > 120 mL/min had insufficient piperacillin concentrations (72). Moreover, around 31% of patients with ARC had underexposures to piperacillin versus 0% in the non-ARC patients (defined using a target of MIC>16 mg/L) (73).

*Meropenem* is a broad spectrum carbapenem antibiotic that exhibits coverage against a wide range of organisms (74). It was reported that around 55% of patients with CL_CR_ > 200 mL/min and received 2 grams every 8 h, had sub-therapeutic concentrations (target 8 mg/mL) (75). A recommended approach to achieve target meropenem concentration in ARC patients is either to increase the dose or administer it via prolonged continuous infusion (76).

It is challenging to administer the right dose to patients with ARC to achieve therapeutic concentrations and avoid any undesirable consequences of sub therapeutic concentrations and treatment failure (67). TDM in such population might be valuable tool to guide the dosing (77), however, there is a clear need for further research to guide specific dosing recommendations in neurocritical care.

### 3.2 Inflammation in neurocritical care

Inflammation is a complex biological response initiated by the immune system following exposure to a range of adverse stimuli in the body, including pathogens, exogenous toxins, ischemia, and tissue injury (78). It is characterized by the activation of immune cells and inflammatory mediators such as cytokines, chemokines, histamines, and acute-phase proteins to coordinate further immune responses, vascular permeability, and tissue remodeling (79). Although localized inflammation is protective against damaging physiological stressors, downstream systemic effects or maladaptive responses can lead to pathophysiological consequences that may impair innate cellular function, compromise vascular integrity, or heighten organ stress (80). Inflammation in neurocritical care patients is consistently linked to worse clinical outcomes, such as neurological deficits, prolonged hospital stays, and higher rates of morbidity and mortality (81).

Many conditions in neurocritical care have been associated with inflammation, including SAH, TBI, IS, ICH, SE and infections of the central nervous system (82–86). For example, patients with SAH have elevated inflammatory markers, resulting in the activation of various inflammatory pathways (81). Animal models of SAH investigated the inflammatory pathways, including the nuclear factor-kappa B (NF-κB) pathway responsible for regulating the expression of pro-inflammatory genes such as tumor necrosis factor alpha (TNF-*α*) and interleukin 6 (IL-6). Activation of the mitogen-activated protein kinase/extracellular signal-regulated kinase (MAPK–ERK) pathway within the MAPK signaling cascade is also implicated in SAH models, elevating the expression of pro-inflammatory cytokines that regulate cellular responses to stress and inflammation (87–89). Similarly, in patients with TBI, neuroinflammation is associated with both the acute and chronic phases of the condition. Immune cell activation occurs at the point of injury, releasing pro-inflammatory cytokines that can disrupt the blood–brain barrier and exacerbate neuronal damage. In the chronic phase, inflammation is linked to ongoing neuronal injury and long-term cognitive and behavioral deficits (90).

Inflammation often complicates neurocritical care by altering drug metabolism and overall clearance, thereby affecting the PK and PD of commonly used therapies in this setting. In Humans and animal models of inflammation, inflammatory cytokines modulate the activity of cytochrome P450 (CYP) drug metabolizing enzymes such as CYP3A4 and CYP2C9, and drug transporters such as P-glycoprotein. This results in decreased drug metabolism, altered drug distribution, and impaired transport across the blood brain barrier (BBB), increasing patient susceptibility to drug toxicity or reduced drug efficacy. Inflammation can also downregulate or alter the conformation of drug targets, such as L-type calcium channels and *β*-adrenergic receptors, diminishing drug binding affinity (91–94). Table 3 summarizes the preclinical evidence of inflammation-induced pharmacotherapy variability in drugs relevant to neurocritical care. For example, nimodipine, a calcium channel blocker utilized to improve outcomes in SAH patients, is metabolized by CYP3A4. Inflammation-induced suppression of this enzyme can lead to reduced nimodipine clearance, causing elevated systemic concentrations and a heightened risk of hypotension in SAH patients (95). Concurrently, the upregulation of P-glycoprotein at the blood–brain barrier seen in preclinical models may limit nimodipine’s ability to penetrate the central nervous system, potentially reducing its therapeutic efficacy despite higher plasma levels (96, 97).

**Table 3:** Preclinical evidence of inflammation-induced pharmacotherapy variability for drugs relevant to neurocritical care (93, 228–236).

Drug	Primary metabolism / Transport/Target	Inflammation impacts	PK changes	PD changes
Nimodipine	CYP3A4 L-type calcium channels	IL-6/IL-2 suppress CYP3A4; upregulation of P-glycoprotein at BBB Inflammatory suppression of calcium channel expression	Decreased clearance, increased systemic levels, decreased CNS penetration	Potential for decreased CNS efficacy despite increased concentrations Potential for decreased CNS efficacy due to reduced receptor binding
Phenytoin	CYP2C9, CYP2C19	Cytokine-mediated suppression of metabolic enzymes	Increased variability in serum levels (increased risk of toxicity)	Unpredictable therapeutic response
Midazolam	CYP3A4	IL-6 suppresses CYP3A4 activity	Drug accumulation; prolonged sedation	Increased sedative effects; delayed emergence from sedation
Levetiracetam	Renal (OCTN1 transporter)	Cytokine effects on transporter activity	Potential changes in CNS distribution	Possible alteration in CNS availability
Propofol	Hepatic (non-specific enzymes), protein binding	Inflammation reduces clearance and alters protein binding	Increased Free drug fraction; prolonged action and risk of toxicity	Increased risk of cardiopulmonary depression
Propranolol	β-adrenergic receptor	Downregulation of β-receptors in inflammatory states	-	Decreased response to beta-blockade
Nicardipine	L-type calcium channels	Inflammatory suppression of calcium channel expression	-	Decreased vasodilatory efficacy

BBB, blood brain barrier; CNS, central nervous system; CYP, cytochrome P450; IL, interleukin.

These interactions emphasize the need for personalized dosing regimens in neurocritical care, considering both the patient’s inflammatory status and the pharmacodynamics of each drug. Systemic inflammation not only worsens the primary injury but also introduces variability in pharmacotherapy, complicating treatment and outcomes. This highlights the importance of targeted anti-inflammatory strategies to optimize therapeutic effectiveness. Future research should focus on developing PK-PD models that address the impact of inflammation, leading to more precise and individualized treatments in neurocritical care.

### 3.3 Neurologic injury and altered drug actions

Neurologic injury itself is a major driver of pharmacotherapy variability in neurocritical care due to its profound effects on both systemic and cerebral physiology as shown in (Table 2) (98–101).

Increased sensitivity to exogenous vasopressin infusion in SAH patients is an example of altered PD of drugs secondary to neurological injury. In critically ill patients with septic shock, exogenous vasopressin administration is infrequently associated with the development of hyponatremia (102). In contrast, Marr et al. reported that the administration of exogenous vasopressin in SAH patients may lead to the development of hyponatremia, which is already the most common electrolyte imbalance encountered after SAH (3). They concluded that vasopressin is an independent predictor of hyponatremia in SAH patients. A possible explanation for this could be that SAH patients have higher serum levels of endogenous vasopressin due to the syndrome of inappropriate anti-diuretic hormone (SIADH), where exogenous vasopressin further enhances the action of the endogenous hormone (103, 104).

Another example is the altered drug absorption secondary to neurological injury-induced gastrointestinal dysfunction. Kranawetter et al. (105) investigated the effect of SAH on the gut function using esomeprazole as a probe. All SAH patients in this study received esomeprazole via feeding tube while the control group swallowed it orally. Median esomeprazole AUC was eight-folds lower in the SAH group compared to the control group (24.8 vs., 208 mg.min/L, respectively, *p- value* < 0.001), suggested significantly reduced oral bioavailability. This aligns with previous results suggesting reduced bioavailability of nimodipine in SAH with increased disease severity (high grade) (106–108). Disease severity itself may be associated with bleeding, gastric reflux, decreased peristalsis, poor perfusion to the splanchnic region potentially contributing to reduced bioavailability of orally administered drugs (105).

In summary, it is important to consider how neurological injury may alter the safety and effectiveness of pre-existing treatments that were previously well-tolerated, as dose adjustments, route change or alternative therapies may be necessary.

## 4 Drug–drug interactions

Drug interactions are common in neurocritical care secondary to polypharmacy, presence of impaired organs function and altered protein binding (109). Interpatient variability, including genetic polymorphisms affecting the activity of multiple CYP enzymes, transporters, and other relevant proteins, also plays a crucial role. Additionally, patient-specific characteristics such as age, sex, race, and social habits can influence drug interactions (109). Mechanistically, these interactions can affect any stage of drug disposition, including any of the PK processes. PD interactions, such as synergism, antagonism, or receptor competition, are also common. Finally, physical incompatibilities, such as the formation of insoluble complexes, can occur, leading to a loss of drug activity (110).

A significant proportion of drug–drug interactions (DDI) occur at the level of hepatic drug metabolism mediated by CYP enzymes (11, 111). Many administered medications are substrates for specific CYPs while simultaneously exhibiting inhibitory or inducing effects on the same or different CYP isoforms. Consequently, the co-administration of drugs impacting CYP enzyme activity frequently results in altered PK of other medications, often necessitating dosage optimization to maintain therapeutic efficacy. For instance, carbamazepine, a commonly used ASM, acts as a potent inducer of CYP3A4 and CYP2B6. Given the broad substrate specificity of CYP3A4, carbamazepine can lead to increased clearance and potentially subtherapeutic concentrations of numerous co-administered drugs (112). Conversely, CYP inhibitors, such as the azole antifungal ketoconazole, certain calcium channel blockers like verapamil, and proton pump inhibitors such as omeprazole, can inhibit the metabolism of their respective substrate drugs (113–115). This inhibition can result in elevated drug plasma concentrations, increasing the risk of adverse drug reactions and toxicity.

Several classes of medications frequently used in neurocritical care warrant careful consideration for potential DDIs, including ASMs (e.g., phenytoin, valproic acid, carbamazepine, benzodiazepines, barbiturates), antimicrobials (e.g., certain beta-lactams and quinolones), calcium channel blockers (e.g., nimodipine, diltiazem, verapamil), sedatives (e.g., dexmedetomidine), and opioids (e.g., fentanyl, morphine) (11).

Therefore, careful assessment and continuous monitoring of DDIs are crucial for reducing pharmacotherapy variability and enhancing patient safety. Adopting a strategy of prioritizing medications with a safer DDI profile, such as drugs primarily eliminated renally with minimal CYP involvement (e.g., levetiracetam compared to other ASMs, certain beta-lactam antimicrobials like piperacillin/tazobactam and cefepime, and the anticoagulant enoxaparin), can contribute to reducing the risk of interactions. However, medication selection in neurocritical care necessitates case-by-case approach to achieve an individualized therapy plan that optimizes therapeutic outcomes while minimizing the risk of DDIs.

## 5 Drug-food interactions

The interaction between drugs and nutrients is a significant concern in hospitalized patients, particularly in the ICU, where the number of prescribed medications is high (116). Drug-food interactions are one of the factors contributing to pharmacotherapy variability and is usually undermined or unrecognized.

The interaction between phenytoin and nutritional feeds is a well-documented example of drug-food interactions that is particularly relevant to neurocritical care. The absorption of phenytoin is influenced by the nutrient composition of the meal. Specifically, high-carbohydrate meals can enhance its absorption, whereas high-protein diets can diminish it (117, 118). Furthermore, binding feeds significantly reduce phenytoin bioavailability. To address this issue, it is recommended to withhold enteral feeding 2 h prior to and following the administration of the drug to ensure optimal bioavailability (118, 119).

The oral administration of certain antimicrobials can be associated with altered bioavailability secondary drug-food interactions. For instance, fluoroquinolones such as ciprofloxacin may form complexes with divalent cations, thereby reducing their absorption and subsequent bioavailability (120, 121). Consequently, it is recommended that ciprofloxacin be taken 1–2 h before meals and vitamin supplementation. Similarly, tetracyclines bind to calcium to form precipitates, which result in sub-therapeutic drug levels, extended hospital stays, and additional economic burdens (122). Furthermore, the bioavailability of azithromycin, a macrolide, is reduced by 43% when taken with food (122).

Nimodipine is another example. The nimodipine monograph notes a 40% reduction in nimodipine peak concentrations and double time to peak concentration, though overall absorption remains consistent, recommending administration with or without meals but consistently (123). In the ICU, crushing tablets for feeding tube delivery leads to erratic concentrations and low bioavailability (95, 106–108, 124). Holding feeds before or after nimodipine dose is impractical for SAH patients who need dosing every two to 4 h. Further research is needed to determine the optimal nimodipine dosing and administration technique to maximize its benefits.

The studies assessing the clinical impact of drug-nutrient interactions are limited, and existing recommendations are based on weak evidence (125). There is a need for well-designed studies. Standardizing drug administration protocols in conjunction with enteral nutrition and developing monitoring methods are crucial steps.

## 6 Co-interventions

Co-interventions, therapies or procedures administered alongside the primary treatment, may contribute to pharmacotherapy variability in neurocritical care. Patients in this setting often undergo a range of concurrent interventions, such as mechanical ventilation, targeted temperature management, continuous renal replacement therapy (CRRT), or the use of vasopressors and sedatives, all of which can influence drug PK and PD. For instance, hypothermia can reduce hepatic enzyme activity and slow drug metabolism, while CRRT can increase drug clearance, particularly for hydrophilic agents with low protein binding. Additionally, fluid resuscitation, altered pH, and hemodynamic instability can further modulate drug distribution and efficacy. These co-interventions can modify drug exposure independently of the primary treatment, contributing to variability in therapeutic outcomes and complicating the interpretation of clinical trial data. Recognizing and adjusting for the impact of co-interventions is essential for accurate dosing, minimizing variability, and ensuring effective, individualized care in neurocritical patients.

### 6.1 Therapeutic plasma exchange

Therapeutic plasma exchange (TPE) is an extracorporeal treatment where blood is withdrawn from a patient’s vein, the plasma is separated, and the blood is subsequently returned to the patient with or without fluid or plasma replacement. TPE is employed in neurocritical care for various conditions, such as Guillain-Barré syndrome and myasthenic crisis. Furthermore, it can be utilized for drug filtration in cases of intoxication.

TPE may affect drug exposure by removing protein-bound drug fractions, which could potentially lead to treatment failure. The PK characteristics of the drug, as well as the specific attributes of the TPE procedure, determine the extent of drug removal during TPE. The volume of distribution (Vd) and plasma protein binding (PPB) are essential in predicting the impact of TPE on drug plasma concentrations (126, 127). A high Vd indicates that the drug is widely distributed throughout the body, resulting in a lower amount of the drug present in the plasma to be removed. Conversely, drugs with a low Vd (0.2–0.3 L/kg) are extensively extracted during the TPE procedure (126–128). PPB defines the fraction of the drug that is bound to plasma proteins and the free unbound fraction remaining. Highly bound drugs (>80%) are readily removed by TPE. For drugs exhibiting multi-compartment PK models, which feature distinctive distribution and elimination phases, the timing of the TPE procedure is critical. Conducting TPE during the drug’s distribution phase results in a higher likelihood of drug removal. The half-life (t_1/2_) and drug clearance are important intrinsic factors for drug removal from plasma. As drug clearance is not always mentioned in monographs, t_1/2_ serves as a surrogate marker for clearance (127–130). TPE-specific factors include the timing of TPE initiation, procedure duration, exchanged plasma volume, and frequency of the procedure. One TPE session typically lasts 2 to 3 h, making drugs with a t_1/2_ longer than 2 h more liable to extraction. The number of plasma volume exchanges correlates directly with the percentage of plasma components removed. The number of TPE sessions impacts drug removal, with most of the drug being removed in the first session, followed by a decrease in the extraction percentage in subsequent sessions due to the exponential nature of drug removal (127, 131).

**Figure 3:**
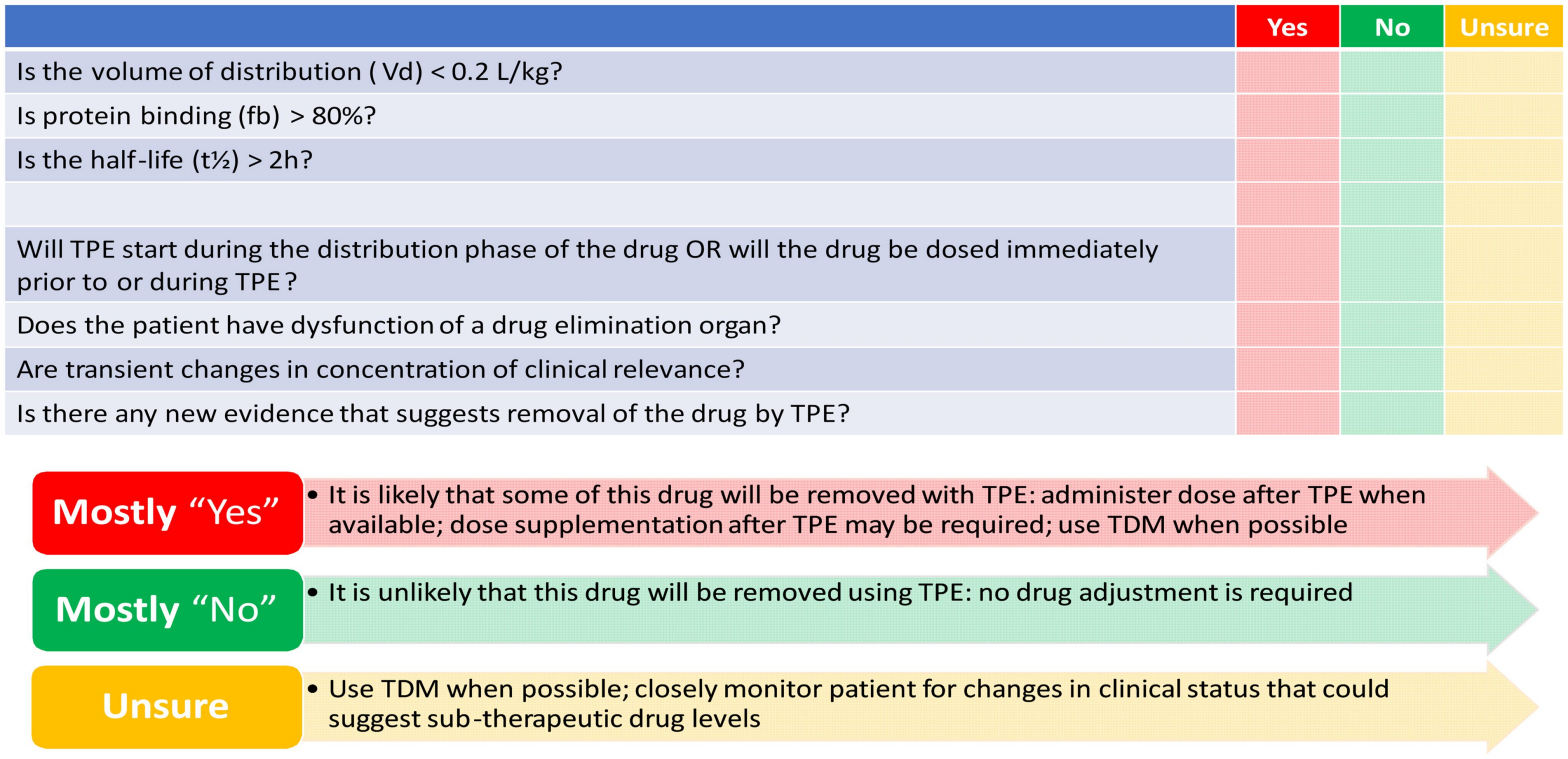
Checklist to determine how likely drugs are removed by therapeutic plasma exchange (TPE). Mahmoud et al. (127) reproduced with permission from Springer Nature.

Evidence reporting drug removal by TPE is scarce; however, utilizing the drug PK characteristics can help estimate the likelihood of drug extraction by TPE. Figure 3 depicts a conceptual proposed tool to determine how likely drugs are removed by TPE based on the PK characteristics of the drug and the current evidence at the time of the study (127). However, this developed tool needs to be validated in future research.

### 6.2 Renal replacement therapies

Renal replacement therapies (RRTs) are not uncommon in neurocritical care (132). Neurological conditions often lead to systemic inflammatory responses and hemodynamic instability, predisposing patients to acute kidney injury (AKI) and necessitating RRT to maintain electrolyte balance, remove metabolic waste, and control fluid overload (133). In this context, RRT serves not only to support renal function but also to optimize neurological recovery by preventing complications like cerebral edema and electrolyte-induced seizures (134–136). The primary RRT modalities used include CRRT, preferred for hemodynamically unstable patients due to its gradual fluid and solute removal, intermittent hemodialysis (IHD), suitable for stable patients requiring efficient solute removal over a shorter time period, and peritoneal dialysis (PD), less common in acute neurocritical care but a possible alternative in some cases (135, 137, 138).

*CRRT* is a form of extracorporeal blood purification employed in hemodynamically unstable critically ill patients, where IHD is poorly tolerated (139). It operates on the principles of convection and diffusion, continuously removing solutes and fluids across a semipermeable membrane via a slow, controlled process (139). Indications include AKI complicated by hemodynamic instability, severe electrolyte imbalances, and conditions necessitating strict fluid management, such as cerebral edema (140, 141). The gradual nature of CRRT minimizes rapid shifts in solute concentrations and intravascular volume, thus reducing the risk of cerebral perfusion pressure fluctuations and elevated intracranial pressure (141). However, CRRT can potentially lead to unintended consequences, including the removal of certain medications, necessitating careful drug dosing adjustments (142). Therefore, subtherapeutic levels of medications are frequently encountered, arising from the combined effects of altered PK in critically ill patients and the extracorporeal clearance provided by CRRT. Antimicrobials (e.g., vancomycin, meropenem and piperacillin-tazobactam) and ASMs (e.g., levetiracetam) demonstrate significant susceptibility to CRRT mediated removal (143). These drugs share PK characteristics, including low protein binding, small volumes of distribution and/or significant renal clearance (144). Consequently, initial recommended dosing regimens often result in subtherapeutic drug levels, potentially compromising therapeutic efficacy and increasing the risk of treatment failure. PK studies are increasingly focused on optimizing drug dosing during CRRT (143, 145–147).

*IHD* is an another blood purification technique that removes waste products and excess fluids over a shorter period, typically 3–4 h (139). It relies primarily on diffusion to clear solutes across a semipermeable membrane, driven by a concentration gradient between the blood and dialysate (139). In neurocritical care, IHD can be utilized when rapid correction of electrolyte imbalances or significant fluid removal is needed, provided the patient can tolerate the hemodynamic shifts associated with the procedure. However, IHD can cause rapid changes in blood volume and electrolyte concentrations, potentially leading to hypotension and disequilibrium syndrome (neurological symptoms due to rapid solute shifts), potentially resulting in increased intracranial pressure (148, 149). Medications, especially those with low volumes of distribution and low protein binding may be significantly cleared during IHD, necessitating careful monitoring and post-dialysis dosing adjustments to maintain therapeutic levels and prevent neurological complications. Similar to CRRT, significant research has been dedicated to optimizing medication dosing regimens in IHD (146).

### 6.3 Therapeutic hypothermia

Therapeutic hypothermia is a treatment strategy that lowers the body temperature intentionally to 32–34°C over a period of 12–24 h (150). It is used in certain conditions such as cardiac arrest, ischemic stroke and traumatic brain injury to limit or restore brain damage (150–153). It has a neuroprotective function as it decreases the brain damage after reduced blood flow (154). There are several hypothesized mechanisms by which therapeutic hypothermia could reduce brain injury. For example, it reduces the metabolic rate by 6–8% per 1°C decrease in the temperature which in turn reduces the brain demand for oxygen. Moreover, it suppresses inflammation, as excessive and continuous inflammation could lead to further brain damage (155). Conversely, some randomized controlled trials did not show any outcome differences if therapeutic hypothermia was applied (156, 157). Therapeutic hypothermia is a three phases process: induction, maintenance and re-warming (155). However, each of these phases carries its risks, and close monitoring of the patient is important. In the induction phase, immediate risks such as electrolyte disturbance, hyperglycemia and shivering could result. On the other hand, monitoring of nosocomial infections and pressure ulcers are important in the maintenance phase. And the rewarming phase should be done very slowly to avoid again electrolytes disturbances and risks of hypoglycemia (155). Therapeutic hypothermia slows down all the processes inside the body which in turn alters the PK and PD of some drugs variably between patients. From the PK point of view, it mainly affects the drugs metabolism and results in increasing the drug concentrations which in turn leads to prolonged response (158). And from the PD aspects it could affect target sensitivity (158).

ASMs such as phenytoin and phenobarbital could be affected by hypothermia (159, 160). A study examined phenytoin PK during and after mild hypothermia (34°C), found that phenytoin metabolism is inhibited by hypothermia. Additionally, phenytoin concentrations were higher during hypothermia compared to concentrations after hypothermia. In addition, there was an 180% increase in AUC and 67% decrease in phenytoin clearance (159). This should be carefully monitored given that phenytoin is a drug with a narrow therapeutic range and any increased serum concentrations could lead to toxicity.

Sedatives and analgesics are commonly used in neurocritical care and therapeutic hypothermia could also impact their disposition in the body. To illustrate, it has been shown that hypothermia (30°C) significantly resulted in increased morphine concentrations in the plasma and cerebrospinal fluid. Moreover, it increased the mean residence time and lowered its clearance significantly (161). Hypothermia also increases the sensitivity to morphine, which could expose the patient to toxicity risks. As such, hypothermia has dual effects on morphine disposition in the body and may lead to morphine toxicity. Additionally, there was a significant decrease in the metabolism of midazolam in healthy volunteers treated with hypothermia. There is a positive correlation between body temperature and inter-compartmental clearance of midazolam, with an 11.1% decrease in clearance for each degree Celsius decrease in temperature (162).

Therapeutic hypothermia has the potential to not only limit cerebral damage but also impact the entire body. Consequently, it is essential to carefully monitor patients undergoing this treatment, and adjustments to medication dosages may be required.

### 6.4 Fluid resuscitation

Fluid resuscitation, a common intervention in neurocritical care, significantly contributes to pharmacotherapy variability by altering the PK of many drugs. Aggressive fluid administration can expand the extracellular fluid volume, leading to dilutional effects and increased volume of distribution, particularly for hydrophilic drugs such as beta-lactam antibiotics (163, 164). This can result in lower plasma drug concentrations and potentially subtherapeutic effects unless dosing is appropriately adjusted (164). Changes in renal perfusion and function caused by fluid resuscitation may also modify drug clearance, either increasing elimination in hyperdynamic states (e.g., ARC) or reducing it in cases of fluid overload and renal impairment (16). These changes necessitate close therapeutic drug monitoring and dose adjustment to ensure effective and safe pharmacotherapy in neurocritical care patients.

## 7 Practice variations

Practice variation in neurocritical care significantly impacts pharmacotherapy variability and clinical outcomes. Differences in institutional protocols, clinician preferences, and resource availability often lead to inconsistent prescribing practices, affecting the quality and uniformity of care. For instance, variations in the choice, dosing and administration of sedatives, ASMs, antimicrobials or anticoagulants can result in diverse pharmacological responses and safety profiles. This inconsistency can lead to underdosing, resulting in therapeutic failure, or overdosing, increasing the risk of adverse drug reactions. Reducing practice variation is critical to optimizing pharmacotherapy and improving outcomes in neurocritical care. Standardized protocols, enhanced communication across multidisciplinary teams, and the integration of decision-support tools into clinical workflows can minimize variability and ensure consistent application of best practices. Such measures, combined with ongoing education and research, can help mitigate the impact of practice variation, fostering more reliable and equitable patient outcomes. In this section we present two examples from our research, highlighting the impact of practice variations on patient outcomes.

### 7.1 Nimodipine administration techniques and formulations

An example of how practice variations could contribute to pharmacotherapy variability is the variability in nimodipine administration in SAH. Nimodipine is a dihydropyridine calcium channel blocker, characterized by being the only member of this class to cross the blood brain barrier and act on the cerebral vascular smooth muscles. Nimodipine inhibits L-type voltage gated calcium channels, hindering the calcium ions influx and hence a vasodilator effect (165, 166). Nimodipine was found to improve outcomes in SAH patients, therefore guidelines recommend that all SAH patients receive a fixed dose of oral nimodipine administered as 60 mg every 4 h for 21 days from ictus (36, 167). To date, nimodipine is the only drug approved for this indication.

**Figure 4:**
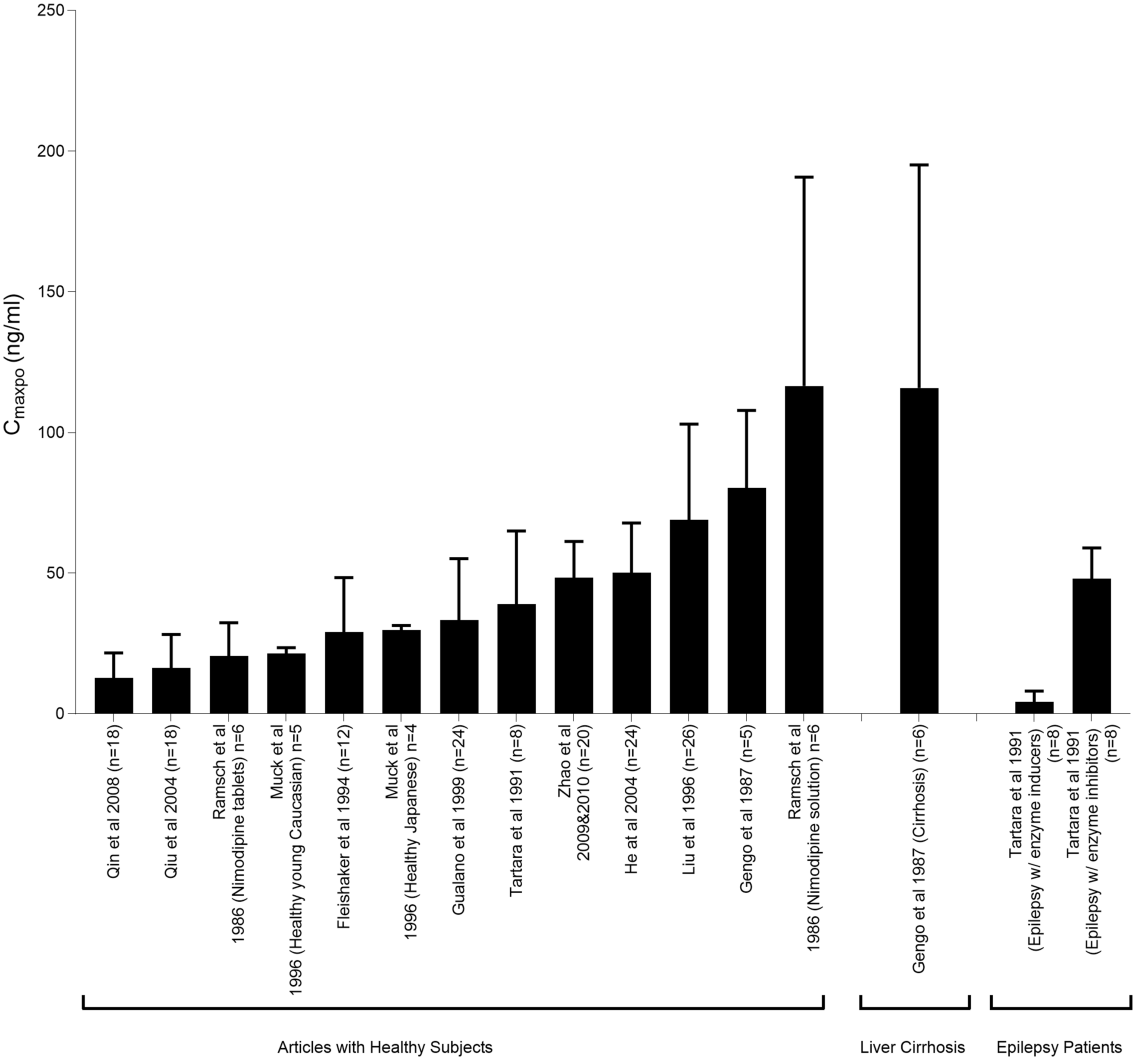
Peak plasma nimodipine concentrations following oral administration (CmaxPO) of a single 60-mg nimodipine dose in healthy individuals and patients with liver cirrhosis and epilepsy. This figure illustrates the pharmacokinetic variability of nimodipine across studies. Mahmoud et al. (97) reproduced from Springer Nature under a creative commons attribution-non commercial 4.0 international license.

Many studies have indicated variability in plasma concentrations of nimodipine, potentially resulting in pharmacotherapy variability (Figure 4). These variations have led to questions regarding whether all patients are receiving the optimal benefit from the administered dose (95, 106, 107, 168). Multiple factors could contribute to nimodipine pharmacotherapy variability such as: age, sex, comorbid conditions, drug–drug interactions, disease severity on admission, genetic polymorphisms, and nimodipine formulations and administration techniques (97). Practice variations in the administration techniques may result in differences in patient outcomes. In a single-center retrospective study comparing the outcomes of administering nimodipine tablets orally (PO) to conscious patients versus delivering the crushed tablet through a feeding tube (FT) to dysphagic, unconscious, or mechanically ventilated patients. The study found that patients who received the crushed tablet via the FT had a higher prevalence of moderate to severe vasospasm and DCI following adjustment for disease severity (124). A multicenter retrospective study conducted across North America included 727 patients from 21 hospitals to compare different nimodipine formulations administered enterally in terms of efficacy and safety. Various oral dosage forms were tested since the oral tablet is the only dosage form marketed in Canada, while capsules and oral solution are available in US institutions. For unconscious, mechanically ventilated, or dysphagic patients, tablets were crushed and administered through FT. Similarly, capsule contents were withdrawn from the gelatin shell using a syringe, either by pharmacists or nurses at the bedside, followed by emptying the syringe content into the FT. Thirty one percent of the patients included in the study developed DCI. The highest prevalence was among the group receiving the crushed tablet via the FT followed by the group receiving the liquid withdrawn from the capsule at bedside (108). From both studies, it is plausible to say that different formulations/administration techniques may not be equivalent. Factors such as differences in excipient formulations, inconsistencies in medication delivery due to institutional practices, and altered drug bioavailability may contribute at least in part to the observed differences (108, 124).

### 7.2 Herpes encephalitis and acyclovir dosing

Herpes encephalitis is a fatal viral infection caused by viruses of the *Herpesviridae* family specifically herpes simplex virus and varicella zoster virus. It has high mortality rate of 70% without treatment, however, even with treatment the mortality rate is 20% (169, 170). Herpes encephalitis diagnosis is confirmed through polymerase chain reaction (PCR) testing of the cerebrospinal fluid to detect the presence of the virus (171). Given the severity of herpes encephalitis, it is crucial to start adequate antiviral therapy as early as possible to improve the morbidity and mortality (172, 173).

Acyclovir is the standard treatment of herpes encephalitis, given intravenously as 10 mg/kg every 8 h for 14–21 days (174, 175). It was first discovered in 1974 to treat cutaneous and genital herpes infection (176, 177); however, in 1994 it was recommended to be started in all patients with suspected encephalitis (172). High acyclovir concentrations in the body could lead to acyclovir nephrotoxicity and neurotoxicity (178). Acyclovir nephrotoxicity is observed as AKI due to precipitation of acyclovir crystals in the kidney tubules (178). Acyclovir induced neurotoxicity observed as hallucination, confusion and other neurological symptoms that also mimic herpes encephalitis (179).

There is a wide practice variations on what body weight to be used in calculating the dose specifically in obese patients (180). An abstract published in 1991, recommended that clinicians should use ideal body weight in calculating the dose to be given to obese patients (181). However, a pharmacist survey reported that there is a clear lack of uniformity to agree on a specific body weight to be used in calculating acyclovir dose in obese patients (182). Pharmacists tend to use adjusted body weight, which is the ideal body weight in addition to the water content of the extra fat in the patient, in overweight patients (182, 183). In obese patients, using actual body weight results in high acyclovir plasma concentrations, which may lead to toxicity (184). On the other hand, using ideal bodyweight could result in lower concentrations compared to using actual body weight in non-obese patients (185). There is no evidence reporting the outcomes associated with using different body weight. Few studies suggest that adjusted body weight could be used in calculating acyclovir dose in obese patients. In our comprehensive literature review, we suggested to use adjusted body weight in obese and actual body weight in non-obese (180).

Another important consideration when dosing acyclovir is the kidney function. The dosing of acyclovir in impaired kidney function patient is well reported, however, its dosing in adult patients with ARC is not well studied. A study conducted on pediatrics patients, suggests that patients with ARC (CL_CR_ > 250 mL/min/1.73m^2^) require larger doses of acyclovir to get effective concentrations (186). However, there is a need for larger prospective studies to confirm the dosing proposals based on the main factors affecting acyclovir concentrations which are body weight to be used and kidney function.

## 8 Pharmacogenomics in neurocritical care

Pharmacogenomics, the study of how genetic variations impact drug response, plays a critical role in optimizing medication therapy (187). Neurocritical care patients often require complex treatment regimens involving high-risk medications with narrow therapeutic ranges such as sedatives, ASMs, analgesics, anticoagulants, and antimicrobials. As time is crucial, the proper choice of drugs considering any genetic variations is important to prevent any secondary brain injury and improve outcomes. Practitioners can improve drug safety and effectiveness in neurocritically ill patients by considering the genetics behind drug metabolism, efficacy, and toxicity. Of the three main areas of pharmacogenomics is metabolism, transporters and targets. Pharmacogenomics variations could be at the level of single gene involvement (monogenic) or more complex involving more than one gene (polygenic) (187, 188).

Around 75% of drugs are metabolized by CYP, where genetic variations could greatly result in drugs PK variation (189). Clopidogrel, an antiplatelet agent commonly used in the ICU, is metabolized by CYP2C19. There are 2 genetic variations in the CYP2C19, specifically the 2* and 3* alleles, that lead to reduced drug activation and increased risk of thrombotic events (190, 191). Another example of a drug that CYP genetic variations affect its PK and PD is codeine, an opioid analgesic often prescribed in neurocritical care (192). CYP2D6 polymorphisms could lead to either poor metabolism or extensive metabolism. Poor metabolism of codeine impairs its activation to morphine and subsequently decreases the analgesic effect. On the other hand, ultra rapid metabolism could lead to morphine toxicity (193). Moreover, in sedatives such as midazolam where CYP3A4/3A5 plays a significant role in its metabolism, genetic variations can change drug clearance, necessitating dose adjustments to avoid either prolonged or inadequate sedation (194).

Genetic variations in drug transporters also play a role in pharmacotherapy variability. For example, polymorphisms in the SLCO1B1 gene affect the transport of statins, which can lead to an increased risk of myopathy (195). Moreover, genetic variations in ABCB1, a p-glycoprotein transporter, could influence the transport of ondansetron and therefore it will result in variability in its antiemetic activity (196). Polygenic variations are common given the complexity and the interplay of several genes to drug disposition in the body. Warfarin, an anticoagulant medication, genetic markers like VKORC1 (target receptor) and CYP2C9 (metabolizing enzyme) are critical for optimizing its anticoagulation (197, 198). Another example is phenytoin. CYP2C9 (metabolizing enzyme) and HLA-B (involved in hypersensitivity reaction) genes play a significant role in phenytoin toxicity (199, 200).

The integration of pharmacogenomics into neurocritical care holds great promise for transforming neurocritical care by enabling a precision-based approach to drug therapy, ultimately aiming to improve the outcomes and prevent toxicity of patients with severe neurological illnesses. However, this is limited by the availability and applicability of genetic testing in acute neurocritical care settings because of the time sensitivity of the setting, the cost of the testing and the complexity of pharmacogenomics data. Overcoming these barriers will require multidisciplinary collaboration, the development of enhanced clinical decision-support tools, and continued research to create a solid pharmacogenomics knowledge base for neurocritical care relevant medications.

## 9 Patient characteristics

Patient-specific characteristics such as age, sex, race, body mass index (BMI), socioeconomic status and social history play a critical role in pharmacotherapy variability in neurocritical care. Clinicians are vigilant to take these characteristics into consideration when making treatment decisions especially for drugs that known to cause adverse effects in patients with specific characteristics. However, less attention is given to the PK/PD changes caused by variable patients’ characteristics.

Age-related physiological changes can significantly impact drug metabolism and clearance. It is not only the elderly patients have reduced renal and hepatic function, but also young adults who are admitted with neurological illness such as TBI are highly prone to have ARC (16).

Many factors could contribute to sex-based differences, such as hormonal and genetic factors. Pharmacotherapy variability could be attributed to different enzyme expression, drug transport, and receptor sensitivity among males and females (201). For example, higher prevalence of ARC was observed in males (16).

Understanding the role of race in pharmacotherapy variability in the neurocritical illness presentations and therapy response is crucial to provide the best care to patients. Because of genetic variations in CYP3A5, an enzyme involved in nimodipine metabolism, different populations could have variable drug exposure depending on the enzyme genotype (extensive, normal, intermediate and poor metabolizers) (202).

Body weight and composition affect Vd and clearance of drugs. For drugs with Vd similar to the total body weight, dosing should carefully consider the type of body weight used. Using ideal body weight may result in lower concentrations due to the water content of fat tissues, while using actual body weight could lead to higher, potentially toxic concentrations. Acyclovir is an example of a drug requiring weight-based dosing, where the choice of weight calculation significantly impacts toxicity, especially in obese patients (180).

Social history such as smoking and alcoholism could greatly impact the drugs PK/PD in the body. Chronic alcohol intake is well known for its induction of CYP2E1enzyme which is involved in metabolizing some drugs such as acetaminophen to its hepatotoxic metabolite (203, 204). Gathering such information is important to be considered as one source of variability.

In neurocritical care, where therapeutic windows are limited and treatment responses can vary, it is important to consider patient-specific characteristics to ensure safe and effective pharmacotherapy.

## 10 Limitations, research gaps, and future directions

This review is limited by its narrative format. The absence of a systematic search methodology may result in subjectivity and restricts the evaluation of the quality of the included studies. Additionally, some studies mentioned in this review do not provide high levels of evidence and their results should be interpreted with caution. Significant research gaps persist in understanding pharmacotherapy variability in neurocritical care. Limited studies have systematically characterized these alterations or translated findings into individualized dosing strategies. The use of potentially ineffective or toxic treatments, as well as suboptimal medication dosing, may contribute to poor patient outcomes due to the limited understanding of how neurological injury influences drug action and disposition. There is a need for precision pharmacotherapy research tailored to neurocritical care to optimize patient outcomes and minimize adverse effects. Further research is required to enhance data capture, characterize clinical phenotypes and their impact on pharmacotherapy variability, and identify biomarkers that predict and guide treatment. Moreover, decision-making tools need to be developed to assist clinicians in making timely decisions within the fast-paced environment of neurocritical care. It will be valuable to incorporate pharmacotherapy variability insights into clinical decision support system. For instance, an electronic health record integrated alert for dosing antimicrobials in a patient with high-risk factors for ARC will efficiently help the clinician to optimize the care. The integration of evidence generated from pharmacotherapy variability research with clinical, genomic, metabolomic, and proteomic data, utilizing machine learning, represents the future of precision medicine in neurocritical care. Table 4 summarizes research focus areas relevant to pharmacotherapy variability and precision medicine in neurocritical care.

**Table 4:** Research focus areas relevant to pharmacotherapy variability and precision medicine in neurocritical care.

Research focus	Details
Prioritize research on PK/PD Alterations	Need for systematic and longitudinal studies on neurological injuries’ effects on drug PK and PD
Develop and validate individualized dosing strategies	Translate PK/PD study findings into practical dosing algorithms and guidelines for clinicians
Enhance data capture and integration	Adopt standardized data collection, leverage Electronic Health Records, integrate multi-modal neuromonitoring and “omics” data
Focus on clinical phenotyping	Identify and validate clinically relevant phenotypes in neurocritical care patients and their association with pharmacotherapy variability
Investigate and validate predictive biomarkers	Identify and validate biomarkers that predict drug response in neurocritically ill patients
Develop and implement clinical decision support tools	Create user-friendly decision support systems that integrate patient data and algorithms for pharmacotherapy decisions
Innovative clinical trial designs	Implement innovative clinical trial designs that address pharmacotherapy variability as contributors for heterogeneity of treatment effect such as adaptive clinical trial designs
Implementation research	Uptake the findings of the evidence-based research and implement it to the routine clinical practice Implement medication safety programs with clinical pharmacists

PK, pharmacokinetics; PD, pharmacodynamics.

## 11 Conclusion

Pharmacotherapy variability within neurocritical care introduces additional layers of complexity that may significantly contribute to therapy failure, adverse drug reactions, and setbacks in drug development. By investigating these unique complexities inherent to neurocritical care, we can advance precision pharmacotherapy, ensuring the administration of the appropriate medication to the right patient at the correct dosage regimen. This approach aims to ultimately enhance clinical outcomes in this vulnerable population.

## Clinical implications

- Pharmacotherapy variability, the variability in drug response among and within individuals, complicates management in neurocritical care by potentially contributing to therapy failure, adverse drug reactions, and setbacks in drug development.
- Clinicians should be cognizant of factors that may contribute to pharmacotherapy variability, including comorbid conditions, drug interactions, practice variations, patient characteristics, pharmacogenomics, and co-interventions.
- Augmented renal clearance (ARC) in neurocritical care accelerates the elimination of renally excreted medications, risking subtherapeutic drug levels and poor treatment outcomes. Proper dosing for those with ARC risk factors is essential.
- Systemic inflammation and neurological injury introduce variability in pharmacotherapy, complicating treatment and outcomes. Further research is needed in this area.
- Clinicians should be vigilant of drug–drug and drug-food interactions in neurocritical care and take appropriate measures to mitigate those interactions, such as holding feeds prior to medication administration, using alternate non-interacting medications, and performing therapeutic drug monitoring.
- Co-interventions, such as therapeutic plasma exchange and renal replacement therapies, may reduce the systemic exposure of drugs, potentially leading to treatment failures. Clinicians should consult available evidence to determine the appropriate dosing and measures to mitigate the effects of co-interventions.
- Incorporating pharmacotherapy variability into treatment guidelines is key to reducing practice variation and advancing personalized care.
- Pharmacists serve as invaluable resources in neurocritical care, offering drug expertise that is essential for managing and minimizing pharmacotherapy variability.
- Until decision-making tools become broadly accessible, individualized patient assessment and monitoring are essential to ensure the delivery of optimal care to patients.
